# The association between blood pressure variability (BPV) with dementia and cognitive function: a systematic review and meta-analysis protocol

**DOI:** 10.1186/s13643-018-0811-9

**Published:** 2018-10-15

**Authors:** VARIABLE BRAIN consortium, Phillip J. Tully, Deborah A. Turnbull, Kaarin J. Anstey, Nigel Beckett, Alexa S. Beiser, Jonathan Birns, Adam M. Brickman, Nicholas R. Burns, Suzanne Cosh, Peter W. de Leeuw, Diana Dorstyn, Merrill F. Elias, J. Wouter Jukema, Kazuomi Kario, Masahiro Kikuya, Abraham A. Kroon, Lenore J. Launer, Rajiv Mahajan, Emer R. McGrath, Simon P. Mooijaart, Eric P. Moll van Charante, Michiaki Nagai, Toshiharu Ninomiya, Tomoyuki Ohara, Takayoshi Ohkubo, Emi Oishi, Ruth Peters, Edo Richard, Michihiro Satoh, Sudha Seshadri, David J. Stott, Willem A. van Gool, Tessa van Middelaar, Stella Trompet, Kristy Giles, Phoebe Drioli-Phillips, Umama Aaimir, Frank Connolly, Christophe Tzourio

**Affiliations:** Centre for Men’s Health, School of Medicine, The University of Adelaide, AHMS Building Level 6, Adelaide, SA 5005 Australia

**Keywords:** Blood pressure variability, Hypertension, Dementia, Cognitive impairment, Ambulatory blood pressure monitoring, Systematic review, Meta-analysis, Protocol, Etiology

## Abstract

**Background:**

A body of empirical work demonstrates that wide fluctuations in a person’s blood pressure across consecutive measures, known as blood pressure variability (BPV), hold prognostic value to predict stroke and transient ischemic attack. However, the magnitude of association between BPV and other neurological outcomes remains less clear. This systematic review aims to pool together data regarding BPV with respect to incident dementia, cognitive impairment, and cognitive function.

**Methods:**

Electronic databases (MEDLINE, EMBASE, and SCOPUS) will be searched for the key words blood pressure variability and outcomes of dementia, cognitive impairment, and cognitive function. Authors and reference lists of included studies will also be contacted to identify additional published and unpublished studies. Eligibility criteria are as follows: population—adult humans (over 18 years but with no upper age limit) without dementia at baseline, with or without elevated blood pressure, or from hypertensive populations (systolic blood pressure ≥ 140 mmHg and/or diastolic blood pressure ≥ 90 mmHg or use of antihypertensive drug for hypertension) and from primary care, community cohort, electronic database registry, or randomized controlled trial (RCT); exposure—any metric of BPV (systolic, diastolic or both) over any duration; comparison—persons without dementia who do not have elevated BPV; and outcome—dementia, cognitive impairment, cognitive function at follow-up from standardized neurological assessment, or cognitive testing. Article screening will be undertaken by two independent reviewers with disagreements resolved through discussion. Data extraction will include original data specified as hazard ratios, odds ratios, correlations, regression coefficients, and original cell data if available. Risk of bias assessment will be undertaken by two independent reviewers. Meta-analytic methods will be used to synthesize the data collected relating to the neurological outcomes with Comprehensive Meta-Analysis Version 2.0 (Biostat Inc., Engelwood, NJ).

**Discussion:**

This systematic review aims to clarify whether BPV is associated with elevated risk for dementia, cognitive impairment, and cognitive function. An evaluation of the etiological links between BPV with incident dementia might inform evidence-based clinical practice and policy concerning blood pressure measurement and hypertension management. The review will identify sources of heterogeneity and may inform decisions on whether it is feasible and desirable to proceed with an individual participant data meta-analysis.

**Systematic review registration:**

PROSPERO CRD42017081977

**Electronic supplementary material:**

The online version of this article (10.1186/s13643-018-0811-9) contains supplementary material, which is available to authorized users.

## Background

Dementia is a highly debilitating disease, leading to profound impairments in quality of life, and comes at great individual, familial, societal, and economic cost. Hypertension, especially in mid-life, is a leading modifiable risk factor for dementia and cognitive decline [1, 2]. The majority of dementia cases have a mix of neurodegenerative and vascular-type pathology evident upon autopsy and brain imaging (e.g., β-amyloid in the former, lacunes in the latter) [3, 4]. The potential for antihypertensive drugs to reduce vascular lesion burden in the brain and dementia risk is a common hypothesis. To date, some randomized controlled trials (RCTs) indicate that differences between active treatment and placebo in systolic blood pressure reduction are associated with cognitive outcome [5–7]. However, the evidence has not favored one particular antihypertensive drug class over others [8–10], and there is no clear hypertension management guideline to assist clinicians hoping to mitigate dementia or cognitive impairment [1]. Clearly, there is more to understand about the role of blood pressure in dementia risk.

A line of research in neurology and cardiology suggests that the intra-individual variability across successive blood pressure readings may be important to neurological outcomes of incident and recurrent stroke or transient ischemic attack [11–14]. However, the magnitude of association between such blood pressure fluctuation, known as blood pressure variability (BPV), and other neurological outcomes remains less clear [15–17]. Though incompletely understood, high BPV potentially leads to carotid artery denervation and endothelial damage reducing perfusion in microvascular vessels [18]. In support of BPV’s relevance to brain health, magnetic resonance imaging studies indicate that BPV in mid-life is associated with greater white matter hyperintensity (WMH) burden [17], with WMH a strong predictor of dementia and stroke [19]. Likewise, higher ambulatory BPV is associated with enlarged perivascular spaces in the brain [20], microinfarcts, and cerebral microbleeds [21]. Moreover, associations between BPV and hippocampal volumes have been shown in cross-sectional analysis [21], and atrophy in this brain region of interest possibly explains other clinical observations from high BPV such as cognitive dysfunction [21–23] or decline [24], dementia [25–27], and late-onset depression [28, 29]. Collectively, there is evidence to suggest that BPV may contribute to vascular pathologies in the brain as well as risk for dementia and cognitive impairment.

Prior systematic reviews concerning blood pressure and dementia have predominantly focused on hypertension or antihypertensive drugs [8, 9, 30–37]. Meta-analyses of BPV have been reported for the neurological outcomes of acute stroke and transient ischemic attack [12] and head-ache [38]. Otherwise, only narrative reviews were reported for the potential role of BPV in dementia and cognitive dysfunction [39–42]. Given the reduction in treat-to-target blood pressure level in recent guidelines [43], and uncertainty around the optimal blood pressure for brain health [1], it is imperative to clarify the role of blood pressure and its variability in relation to dementia [44]. A systematic review and meta-analysis pertaining to BPVs’ association with dementia, cognitive impairment, and cognitive function might in turn assist in the design of subsequent epidemiological studies and inform clinicians.

## Methods

### Aims

The proposed review aims to synthesize the evidence base regarding BPV and subsequent dementia or cognitive impairment. The reporting of this protocol conforms to the PRISMA-P guidelines [45] (shown in Additional file 1) and was registered on the PROPSERO database [CRD42017081977] on the 8th of December, 2017 [46]. The full review will conform to the PRISMA guidelines [47]. PJT is guarantor of this review. Updates to this review will be registered on PROSPERO.

### Search strategy

We will identify relevant articles in any language by searching electronic databases from inception including the following: MEDLINE, EMBASE, and SCOPUS. The MEDLINE search strategy is provided in Additional file 2. We will perform a hand search of the reference lists of articles selected to supplement the electronic search. The principal investigators of studies will also be contacted to ascertain unpublished data and determine other studies not yielded by our primary search. The grey literature/unpublished studies will not be searched on an electronic database.

### Eligibility criteria

Population: the population of interest are adult humans (over 18 years but with no upper age limit) from the general population, primary care, or other population, without verified or known dementia at baseline, whom underwent consecutive blood pressure measures. Persons may be with or without elevated blood pressure or from hypertensive populations (systolic blood pressure ≥ 140 mmHg and/or diastolic blood pressure ≥ 90 mmHg or use of antihypertensive drug for hypertension). To be eligible, blood pressure must be quantified by a valid method standardized within studies including office, home, ambulatory, or beat-to-beat measures from which BPV was calculated, or can be determined by published results or clarified by authors. In the case that studies include persons with dementia at baseline, we specify that the association between BPV and incident dementia must be reported separately to be included in the systematic review analyses.

Exposure: any measure of BPV (systolic, diastolic, or both) calculated utilizing any common metric (e.g., standard deviation [SD], average real variability [ARV], coefficient of variation [CV]) for systolic and diastolic BPV [48]. BPV data will be extracted and prioritized in the order of beat-to-beat, 24-h ambulatory blood pressure monitoring (ABPM), awake ABPM measures (versus sleep), office/clinic/casual (also known as visit-to-visit), and home blood pressure monitoring. Instances where the BPV M ± SD data are reported for more than two groups, data will be extracted for the highest BPV versus lowest BPV group (in tertiles, quartiles, quintiles, or deciles) to ensure that only one effect size is analyzed per study and that dissimilar *n = k* groups are pooled in analyses.

Comparator/control: participants without verified or known dementia, and without elevated BPV at baseline from the general population, primary care, or other population. There are no known normative values for elevated and non-elevated BPV, which will likely be reported according to within study cutoffs (e.g., in tertiles, quartiles, quintiles, or deciles).

Outcomes:

1. Dementia—defined as a diagnosis according to a recognized and standardized clinical criteria (e.g., National Institute of Neurological and Communicative Disorders and Stroke and Alzheimer’s Disease and Related Disorders Association [NINCDS-ADRDA], National Institute of Neurological Disorders and Stroke Association-Internationale pour la Recherche en l’Enseignement en Neurosciences [NINDS-AIREN], Diagnostic and Statistical Manual of Mental Disorders [DSM]) or a diagnosis made by a qualified professional (e.g., neurologist, geriatrician, psychiatrist, general physician). Dementia will be considered in any of the following categories: Alzheimer’s disease (AD), vascular dementia (VaD), mixed dementia, dementia unspecified, and other dementia. In studies reporting multiple dementia subtypes, we will initially extract total incident dementia for primary analyses and evaluate subtypes in stratified analyses. When levels of AD diagnoses were provided within the same study, we will prioritize probable AD and exclude possible AD.
2. Cognitive impairment or decline—defined as an objective cognitive impairment or between assessment decline (e.g., 1 SD reduction, reliable change index) or below age-sex appropriate normative data on a standardized test of cognitive function representing memory (episodic memory, semantic memory, or overall memory ability), language (verbal fluency), speed (processing speed), visuospatial abilities, or executive functioning (working memory, reasoning, attention, or overall executive functioning) or global cognition, including the Mini Mental State Examination [49]. Self-reported cognitive decline will not be considered.
3. Cognitive function—defined as cognitive test scores on a standardized test of cognitive function representing memory (episodic memory, semantic memory or overall memory ability), language (verbal fluency), speed (processing speed), visuospatial abilities, or executive functioning (working memory, reasoning, attention, or overall executive functioning) or global cognition, including the Mini Mental State Examination [49]. Self-reported cognitive function will not be considered.

We will assess the primary outcomes as independent outcomes: incident dementia (level 1), cognitive impairment or decline (level 2), cognitive function (level 3), and any dementia or cognitive impairment or decline (combining study data for level 1 and level 2 outcomes).

#### Study design

Only peer reviewed studies in full-text, conference abstract or doctoral dissertations are eligible for this review if published in English [50]. Observational studies designed as longitudinal cohort, case–control study, or database registry and experimental studies designed as RCTs or non-randomized trials will be eligible. Prospective and retrospective studies will also be eligible. We will exclude cross-sectional studies, case series, and case reports.

#### Exclusion

Studies utilizing only patient self-report to determine incident dementia or cognitive impairment, including self-report of a physician’s diagnosis that is not further verified consistent with the definitions listed under study outcomes (levels 1–3), are excluded. Studies reporting dementia secondary to a primary degenerative or neurological condition or insult are excluded (e.g., tumor, infection, traumatic brain injury, Wernicke’s encephalopathy).

### Study selection process

Initially, two reviewers will independently screen titles and abstracts of all the retrieved bibliographic records. Full texts of all potentially eligible records passing the title and abstract screening level will be retrieved and examined independently by the two reviewers according to the abovementioned eligibility criteria. Disagreements at both screening levels (title/abstract and full text) will be adjudicated by discussion with a third reviewer. A PRISMA flow chart will outline the study selection process and reasons for exclusions.

### Data items for collection

After determination of the initial study, eligibility information will be extracted for each study pertaining to study identification (first author, year of publication, country where recruitment took place), study design and characteristics (sample size, duration of follow-up, attrition), characteristics of the population under study (age, sex, education, systolic and diastolic blood pressure, proportion with hypertension, hypercholesterolemia, diabetes, kidney disease, liver disease, stroke, apolipoprotein ε4 polymorphism, coronary heart disease, and heart failure), BPV exposure (methodology or methodologies), dementia adjudication (criteria, subtypes, use of consensus panel, number of endpoints), cognitive testing (full list of cognitive test(s) and their domains), effect size (unadjusted and most adjusted effect size or raw numbers), adjustment for covariates (list of variables), and funding (grant numbers or acknowledgement). Primary outcome data collected will include the type of cognitive outcome, reported either as categorical numbers (numerator and denominator), or the statistical effect size (e.g., risk ratio, hazard ratio) and the 95% confidence interval (CI). These variables will be extracted for all studies by one reviewer, after which the extracted data will be verified by a second reviewer to reduce reviewer errors and bias. All disagreements will be handled by consensus between the two reviewers. Data will be managed at the coordinating center (University of Adelaide).

### Risk of bias

The RTI item bank will be utilized to identify methodological bias among the identified studies at the study-level [51]. The RTI item bank consists of 29 items for evaluating the risk of bias in observational studies, interventions, or exposures. The RTI was developed from an initial pool of 1492 items based on face validity, cognitive, content validity, and interrater reliability testing. The scale has demonstrated interrater reliability. Risk of bias will be independently undertaken by two reviewers, and disagreements resolved by consensus. The RTI item bank is provided in Additional file 3.

### Synthesis of data and summary measures

#### Data synthesis

We will provide a detailed description of the results in both tables and text for all included studies. We will qualitatively describe the studies pertaining to study identification (first author, year of publication, country where recruitment took place), study design and characteristics (observational or experimental, sample size, duration of follow-up), patient population (age, sex), the methods used to quantify BPV, the type of cognitive endpoint (dementia, cognitive impairment, cognitive function), and adjustment for covariates (list of variables).

#### Meta-analysis

We will use Comprehensive Meta-Analysis Version 2.0 (Biostat Inc., Engelwood, NJ) to conduct the meta-analyses. The summary effect measures may include *d* family effect sizes (e.g., hazard ratios, relative risk, odds ratios, Cohen’s *d*) or *r* family effect sizes (e.g., *r*, *β*). When data are available to be pooled together, we will use a random-effects model using the inverse variance method which provides a more conservative estimate of effect size. Where possible, we will aggregate each included study’s cognitive outcome data from the *d* family of effect sizes with the associated 95% CIs. Hazard ratios, relative risk, and odds ratios are presumed to measure the same underlying effect [52] and consensus that these are approximately equivalent for effect sizes less than 2.5 and follow-up less than 20 years [53]. In studies where an effect size is not reported, we will extract the individual cell data and calculate the RR and 95% CI where possible.

The *r* family effect sizes will be converted to the common metric *r* and pooled together with their associated 95% CI.

In the first instance, we will pool together the unadjusted effect sizes for each cognitive outcome (permitting age and sex adjustment). In the second instance, we will pool together the most adjusted effect sizes for each cognitive outcome. Heterogeneity will be evaluated with two measures of between-study heterogeneity: the *I*^2^ statistic and tau (equivalent to SD of pooled *r*). According to the Cochrane Handbook for Systematic Reviews [54], *I*^2^ of 0–60% can be regarded as not important to moderate (0–60%), while *I*^2^ > 60% indicates substantial heterogeneity.

#### Planned subgroup analyses

Subgroup analyses will be performed for the combined cognitive outcome (combination of level 1 dementia and level 2 cognitive impairment or decline data) stratified by sex if possible. In the event that stratified sex analyses is not possible, we will utilize meta-regression to perform analyses adjusted for the percentage of males/females in the total sample, and age (mean or median). Sensitivity analyses will evaluate the effects of age < 50 and > 50 years, or alternatively as mean or median age in meta-regression. This is based on the a priori higher probability for older persons to have dementia and the age-dependent relationship between mid-life blood pressure and dementia [55]. Also, a subgroup analysis will be performed, if possible, stratifying by dementia sub-types of Alzheimer’s disease and vascular dementia.

#### Planned sensitivity analyses

Sensitivity analyses will be performed for the primary cognitive endpoint (combination of level 1 dementia and level 2 cognitive impairment or decline data). The period during which blood pressure was measured may be a source of methodological heterogeneity. We will therefore stratify analyses according to blood pressure measurement intervals as: 24 h or less (e.g., beat-to-beat, 24-h ABPM), short term > 24 h to 1 month, medium term > 1 month to ≤ 12 months, and long term > 12 months. We will also assess general study-level characteristics as potential sources of heterogeneity (1) studies adjusting for reverse causation bias by excluding dementia events occurring in the years of follow-up when BPV was calculated, (2) global region of recruitment, (3) unpublished studies, and (4) length of follow-up. Due to the insidious prodromal phase of dementia, we will also perform a meta-regression based on attrition and mean follow-up time to consider competing risks and survivor bias [56]. Follow-up duration will be dichotomized as short term (up to 5 years) and medium to long term (more than 5 years). This was based on the consensus that exposure to antihypertensive drugs in RCTs have likely been too short (< 5 years) for antihypertensive treatment to positively impact on cognition [57]. We intend to group all studies together initially and then perform sensitivity analyses for different time points (e.g., dementia in the short and long term), if possible.

#### Assessment of publication bias

The test of Egger et al. [58] and the funnel plot will be used to evaluate the presence of publication bias.

#### GRADE framework for quality of evidence

The proposed review will use the Grading of Recommendations Assessment, Development and Evaluation (GRADE) guidelines [59] to determine the quality of evidence and the strength of recommendations. The GRADE guidelines will be applied separately to each of the cognitive endpoints, providing a summary of findings tables with qualitative description as either high, moderate, low, or very low.

## Discussion

This systematic review aims to add to the literature by aggregating data concerning the risk of dementia and cognitive impairment attributable to BPV. Our review will contribute to the literature by clarifying whether BPV, derived from consecutive blood pressure measurements, is associated with these cognitive outcomes. It is well established that hypertension in mid-life is associated with an increased risk for dementia [55]. However, RCTs of antihypertensive drugs have not consistently reduced dementia risk. The findings of our review might therefore serve to clarify the design of future epidemiological and clinical studies. The findings might also potentially inform evidence-based clinical practice and policy regarding blood pressure measurement and hypertension management, especially in older persons at greater risk for cognitive impairment and conversion to dementia.

There are several limitations that will contextualize the findings and generalizability of the proposed review including that high BPV (e.g., upper quintile of the population) is the result of complex interactions between cardiovascular regulatory mechanisms (neural central, neural reflex) and environmental and behavioral factors [18]. As such, we may not be able to identify antecedent risk factors for higher BPV or ways to modulate BPV in order to lessen dementia risk. Similarly, BPV can be dependent on mean blood pressure in some methodologies (e.g., coefficient of variation); thus, it may be difficult to extrapolate the effects of BPV independent from mean blood pressure. The included studies will also potentially measure blood pressure at different intervals ranging from beat-to-beat to long-term visit-to-visit in epidemiological studies, which could introduce methodological heterogeneity. Limitations will also relate to the adjudication of dementia outcomes with varying levels of validity and heterogeneity. The ability for correct adjudication of dementia outcomes will be invariably related to the age of participants and the length of follow-up. This limitation is important in our proposed review’s context since we are largely assessing etiological links between BPV and dementia. Limitations of the original studies may also include between study heterogeneity and high risk of bias that will potentially limit the conclusions drawn. The proposed systematic review is likely to be limited by publication bias of only significant findings, given the relatively recent interest in BPV since Rothwell and colleagues seminal work on this topic [11–13]. Moreover, as the proposed review will include only English language studies, the generalizability of the findings to studies published in other languages and other healthcare settings is limited.

In conclusion, given that there is still uncertainty around the optimal blood pressure for brain health [1], it is imperative to clarify the role of BPV on cognitive outcomes. The proposed review will help in summarizing the available evidence, and the findings may have implications for clinical practice and policy concerning blood pressure measurement and hypertension management. The review will identify sources of heterogeneity and may inform decisions on whether it is feasible and desirable to proceed with an individual participant data meta-analysis.

## Additional files

Additional file 1: PRISMA-P checklist of reporting items for this systematic review protocol. This file shows the page number for each item on the PRISMA-P checklist. (PDF 372 kb)

Additional file 2: Table showing the search strings for MEDLINE. This table shows the search string for the systematic review for the MEDLINE database utilized in this review. This search string will be adapted for EMBASE and SCOPUS. (PDF 440 kb)

Additional file 3: RTI risk of bias item bank. This table shows each of the items of the RTI item bank used in our study. Each of the studies selected for full-text review will be scored to these items by two reviewers. (PDF 454 kb)

## Abbreviations

ABPM: Ambulatory blood pressure monitoring
BPV: Blood pressure variability
CI: Confidence interval
DSM: Diagnostic and Statistical Manual of Mental Disorders
GRADE: Grading of Recommendations Assessment, Development and Evaluation
HR: Hazard ratio
NINCDS-ADRDA: National Institute of Neurological and Communicative Disorders and Stroke and Alzheimer’s Disease and Related Disorders Association
NINDS-AIREN: National Institute of Neurological Disorders and Stroke Association-Internationale pour la Recherche en l’Enseignement en Neurosciences
OR: Odds ratio
RR: Risk ratio
WMH: White matter hyperintensity
