# Supplementary material for: The association between blood pressure variability (BPV) with dementia and cognitive function: a systematic review and meta-analysis protocol
Source: Syst Rev. 2018 Oct 15;7:163. doi: 10.1186/s13643-018-0811-9 (PMC6190539; doi:10.1186/s13643-018-0811-9)
Supplement: Supplementary file 2 — Table showing the search strings for MEDLINE. This table shows the search string for the systematic review for the MEDLINE database utilized in this review. This search string will be adapted for EMBASE and SCOPUS. (PDF 440 kb) [file 13643_2018_811_MOESM2_ESM.pdf]

Additional file 2 Search String

MEDLINE
“blood pressure variability” OR “visit-to-visit” OR “ambulatory blood pressure monitoring” OR “24-hour blood pressure monitoring” OR “home blood pressure monitoring” AND “dementia” OR “Alzheimer’s disease” OR “vascular dementia” OR “cognitive impairment” OR “Mini Mental State” OR “cognitive function” OR “cognitive testing” OR “neuropsychological testing” OR “memory”.
