# Supplementary material for: The association between blood pressure variability (BPV) with dementia and cognitive function: a systematic review and meta-analysis protocol
Source: Syst Rev. 2018 Oct 15;7:163. doi: 10.1186/s13643-018-0811-9 (PMC6190539; doi:10.1186/s13643-018-0811-9)
Supplement: Supplementary file 3 — RTI risk of bias item bank. This table shows each of the items of the RTI item bank used in our study. Each of the studies selected for full-text review will be scored to these items by two reviewers. (PDF 454 kb) [file 13643_2018_811_MOESM3_ESM.pdf]

Additional File 3. RTI Risk of Bias Item Bank

Risk of bias item	Description	
1. Retrospective/ prospective	**Is the study design prospective, retrospective, or mixed?** *[Abstractor: Prospective design requires that the outcome has not occurred at the time the study is initiated and information is collected over time to assess relationships with the outcome (and includes nested case-control studies). Mixed design includes case-control or cohort studies in which one group is studied prospectively and the other retrospectively. A retrospective design analyzes data from past records. The question is not applicable to cross-sectional studies.]*	Prospective .... Mixed .... Retrospective .... Cannot determine/not applicable .... **Explanation for rating:**
2. Inclusion/ exclusion criteria stated	**Are critical inclusion/exclusion criteria clearly stated (does not require the reader to infer)?** *[Principal Investigator (PI): Provide direction to abstractors by listing individual criteria of a priori significance and minimal requirements for criteria to be considered "clearly stated." Include this question to identify specific inclusion/exclusion criteria that should be consistently recorded across studies]* *[Abstractor: Use "Partially" if only some criteria are stated or if some criteria are not clearly stated (corresponding to directions provided by the PI). Note that studies may describe inclusion criteria alone (i.e., include x), exclusion criteria (i.e., do not include x), or a combination of inclusion and exclusion criteria.]*	Yes .... Partially: some, but not all, criteria stated or some criteria not clearly stated .... No .... **Explanation for rating:**

3. Inclusion/ exclusion criteria reliable	**Are the inclusion/exclusion criteria measured using valid and reliable measures?** *[PI: Separately specify each criterion that abstractors should consider based on its relevance to study bias. It is unlikely that all criteria will need to be evaluated in relation to this question. Provide direction to abstractors on valid and reliable measurement of each criterion that is to be considered. For example, prior exposure or disease status is a frequent inclusion/exclusion criterion, particularly in inception cohorts. Subjective measures based on self-report tend to have lower reliability and validity than objective measures such as clinical reports and lab findings.*	Yes ....<br>No ....<br>Cannot determine; measurement approach not reported ....<br>**Explanation for rating:**
4. Inclusion/ exclusion criteria uniform	***Did the study apply inclusion/exclusion criteria uniformly to all comparison groups/arms of the study?*** *[PI: Drop question if not relevant to entire body of evidence (e.g., all case-series, singlearm studies).]*	Yes ....<br>Partially: some, but not all criteria, applied to all arms or not clearly stated if some criteria are applied to all arms ....<br>No ....<br>Cannot determine: article does not specify ....<br>Not applicable: study has only one arm and so does not include comparison groups ....<br>**Explanation for rating:**
5. Recruitment across groups	**Was the strategy for recruiting participants into the study the same across study groups/arms of the study?** *[PIs: This question is likely to be more relevant for prospective or mixed designs than retrospective designs. Drop question if not relevant to entire body of evidence (e.g., all studies generally have only one arm).]*	Yes ....<br>No ....<br>Cannot determine ....<br>Not applicable: one study group/arm ....<br><br>**Explanation for rating:**

6. Statistical power	***Was the sample size sufficiently large to detect a clinically significant difference of 5% or more between groups in at least one primary outcome measure?*** *[PI: Specify a different percent, if clinically relevant for each outcome of interest. Question relates to precision; reviewers whose evaluation of quality is limited to considerations of systematic error or risk of bias (not random error/precision) need not include this question. Reviewers who include both precision and systematic error in their evaluation of quality but rely on meta-analysis for pooled estimates need not include this question. PIs who choose to include considerations of precision in their assessment may include the question, but should be aware of the need for collaboration between clinical and statistical expertise in determining the threshold for a clinically adequate sample size.]*	Yes<br>..<br>No<br>...<br>**Explanation for rating:**
7. Detail of exposure	***What is the level of detail in describing the intervention or exposure?*** *[PI: Specify which details need to be stated (e.g., intensity, duration, frequency, route, setting, and timing of intervention/exposure). For case-control studies, consider whether the condition, timing, frequency, and setting of symptoms are provided in the case definition. PI needs to establish criteria for high, medium, or low response.]*	High: very clear, all PI-required details provided<br>Medium: somewhat clear, majority of PI required details provided<br>....................................<br>Low: unclear, many PI-required details missing<br>..<br><br>**Explanation for rating:**
8. Specification of outcomes	**Are the important outcomes pre-specified by the researchers? Do not consider**	Yes<br>..

	**harms in answering this question unless they should have been pre-specified.** *[PI: This question can be asked for all outcomes together or replicated for each event. Each adverse event of interest should be specified for abstractors. Relevant source information includes all study data, including what may have been established in relation to an initial randomized controlled trial. Drop question if not relevant (e.g., primary outcome for case-control studies).]*	Partially .... No .... Not applicable ....<br><br>**Explanation for rating:**
9. Appropriate comparison group	**Is the selection of the comparison group appropriate, after taking into account feasibility and ethical considerations.** *[PI: Provide instruction to the abstractor based on the type of study. Interventions with community components are likely to have contamination if all groups are drawn from the same community. Interventions without community components should select groups from the same source (e.g., community or hospital) to reduce baseline differences across groups. For case-control studies, controls should represent the population from which cases arose; that is, controls should have met the case definition if they had the outcome.]*	Yes .... No .... Cannot determine or no description of the derivation of the comparison group .... Not applicable: study does not include a comparison group (case series, one study arm) ...<br><br>**Explanation for rating:**
10. Attempt to Balance	**Any attempt to balance the allocation between the groups (e.g., through stratification, matching, propensity scores).** *[PI: This is most likely to be used in case-control study designs. Drop if not relevant to the body of evidence.]*	Yes or study accounts for imbalance between groups through a post hoc approach such as multivariate analysis .... No or cannot determine .... Not applicable: study does not include a

		comparison group (case series or one study arm)<br><br>**Explanation for rating:**
11. Adjustment for unintended exposure	**Did researchers isolate the impact from a concurrent intervention or an unintended exposure that might bias results, e.g., through multivariate analysis, stratification, or subgroup analysis?** *[PI: specify interventions or exposures for abstractors.]*	Yes ....<br>Partially ....<br>No or do not know: concurrent intervention or Unintended exposure is not described) ....<br>Not applicable: no concurrent interventions or unintended exposures likely ....<br>**Explanation for rating:**
12. Variation in execution of protocol	**Did execution of the study vary from the intervention protocol proposed by the investigators and therefore compromise the conclusions of the study?** *[PI: Consider intensity, duration, frequency, route, setting, and timing of intervention/exposures. Drop if not relevant for body of literature.]*	Yes ....<br>Partially ....<br>No ....<br>Cannot determine ....<br>Not applicable: not an intervention study ....<br><br>**Explanation for rating:**
13. Blind outcomes assessment	**Were the outcome assessors blinded to the intervention or exposure status of participants?** *[PI: There may be circumstances where clinical evaluators cannot be blinded to exposure status. Drop if not relevant to the body of literature.]*	Yes ....<br>No ....<br>Not applicable: assessor cannot be blinded ....<br><br>**Explanation for rating:**
14. Exposures assessed using valid	**Are interventions/exposures assessed using valid and reliable measures, implemented consistently across all study participants?** *[PI: Important*	Yes ....<br>No ....

and reliable measures	*measures may be listed separately. PI may need to establish a threshold for what would constitute acceptable measures based on study topic. When subjective or objective measures could be collected, subjective measures based on selfreport may be considered as being less reliable and valid than objective measures such as clinical reports and lab findings. Replicate question when needed.]*	Cannot determine or measurement approach not reported .... **Explanation for rating:**
15. Outcomes assessed using valid and reliable measures	**Are outcomes assessed using valid and reliable measures, implemented consistently across all study participants?** *[PI: Primary outcomes should be identified for abstractors and if there is more than one, they may be listed separately. Also, identify any relevant secondary outcomes and harms. Subjective measures based on self-report tend to have lower reliability and validity than objective measures such as clinical reports and lab findings. Note for case-control studies: consider whether the ascertainment of cases was independent of exposure.]*	Yes .... No .... Cannot determine or measurement approach not reported .... **Explanation for rating:**
16. Equality of length of f/u	**Is the length of follow-up the same for all groups?** *[For case-control studies, are cases and controls matched on length of followup? Abstractor: When follow-up was the same for all study participants, the answer is yes. If different lengths of follow-up were adjusted by statistical techniques, (e.g., survival analysis), the answer is yes. Studies in which differences in follow-up*	Yes .... No or cannot determine .... Not applicable: cross-sectional or only one group followed over time .... **Explanation for rating:**

	were ignored should be answered no.]	
17. Length of f/u adequate	**Is the length of time following the intervention/exposure sufficient to support the evaluation of primary outcomes and harms?** *[PI: Primary outcomes (including harms) should be identified for abstractors. Important measures may be listed separately. Abstractors should be provided with specific criteria for sufficient length of follow-up based on prior research or theory. Drop if entire body of evidence is cross-sectional or if minimal length of follow-up period is specified through inclusion criteria.]*	Yes ....<br>Partially: some primary outcomes are followed<br>for a sufficient length of time ....<br>No ....<br>Cannot determine ....<br>Not applicable: cross-sectional ....<br><br>**Explanation for rating:**
18. High attrition	**Did attrition from any group exceed [x] percent?** *[PI: Attrition is measured in relation to the time between baseline (allocation in some instances) and outcome measurement for both retrospective and prospective studies and could include data loss from crossover. Attrition rates may vary by outcome and time of measurement. Specify the criterion to meet relevant standards for the topic. Specify measurement period of interest, if repeated measures. Cochrane standard for attrition is 20 percent for shorter term (<1 year) and 30 percent for longer term (≥ 1 year). Drop if entire body of evidence is cross-sectional]*	Yes ....<br>No ....<br>Cannot determine: includes retrospective designs not stating number eligible at baseline ....<br>Not applicable: cross-sectional ....<br><br>**Explanation for rating:**
19. Attrition difference	**Did attrition differ between groups by more than 20 percent?** *[PI: If appropriate, modify difference criterion to meet relevant standards for the topic. Attrition rates may vary by*	Yes ....<br>No ....<br>Cannot determine: includes retrospective

	outcome and time of measurement. Drop if entire body of evidence is cross-sectional or case series.]	designs not stating number eligible at baseline ....<br>Not applicable: cross-sectional or only one group followed—case series, one-arm study ....<br><br>**Explanation for rating:**
20. Baseline differences controlled	**Does the analysis control for baseline differences between groups?** *[PI: Drop if entire body of evidence is case series or case control. Define adequate control. List critical baseline differences that need to be controlled.]*	Yes ....<br>No ....<br>Insufficient reporting to be able to determine ......<br>Not applicable: only one group, no comparison group (case series), or case-control study, no difference in measured baseline characteristics ...<br>**Explanation for rating:**
21. Measurement of confounding variables reliable	**Are confounding and/or effect modifying variables assessed using valid and reliable measures across all study participants?** *[PI: Some characteristics may require that sources for establishing their validity and/or reliability be described or referenced. If so, provide instruction to abstractors.]*	Yes ....<br>No ....<br>Cannot determine or source for measures not reported ....<br>Not applicable: no confounders or effect modifiers included in the study ....<br><br>**Explanation for rating:**
22. Confounding variables in design/ analysis	**Were the important confounding and effect modifying variables taken into account in the design and/or analysis (e.g., through matching, stratification, interaction terms, multivariate analysis, or other statistical adjustment)?** *[PI: Provide instruction to abstractors on adequate adjustment for confounding and testing for effect modification.]*	Yes ....<br>Partially: some variables taken into account or<br>adjustment achieved to some extent ....<br>No: not accounted for or not identified ....<br>Cannot determine ....<br><br>**Explanation for rating:**

23. Sensitivity analysis for loss to f/u	**In cases of high loss to follow-up (or differential loss to follow-up), is the impact assessed (e.g., through sensitivity analysis or other adjustment method)?**	Yes .... No .... Cannot determine .... Not applicable: no loss to follow-up or loss to follow-up was not considered to be high, crosssectional study, or case-control study selected on outcome .... **Explanation for rating:**
24. Primary outcomes missing	**Are any important primary outcomes missing from the results?** *[PI: Identify all primary outcomes, including timing of measurement, that one would expect to be reported in the study.]*	Yes .... No .... Cannot determine .... **Explanation for rating:**
27. Appropriate statistics for outcome	***Are the statistical methods used to assess the main harm or adverse event outcomes appropriate to the data?*** *[Abstractor: Question relates to precision and may not be relevant for systematic reviews that are able to pool data. The statistical techniques used must be appropriate to the data and take into account issues such as controlling for dose-response, small sample size, clustering, rare outcomes, and multiple comparisons. In normally distributed data, the standard error, standard deviation, or confidence intervals should be reported. In non-normally distributed data, inter-quartile range should be reported.]*	Yes .... Partially .... No .... Cannot determine .... **Explanation for rating:**

28. Appropriate interpretation	**Are results believable taking study limitations into consideration?** *[Abstractor: This question is intended to capture the overall quality of the study. Consider issues that may limit your ability to interpret the results of the study. Review responses to earlier questions for specific criteria.]*	Yes .... Partially .... No .... **Explanation for rating:**
29. Funding	**Is the source of funding identified?** *[PI: The relevance of this question will depend upon the topic. This question may be modified to identify particular sources of funding (e.g., industry, government, university, or foundation funding).]*	Yes .... No .... **Explanation for rating:**

RTI item bank [1]

1. Viswanathan M, Berkman ND: **Development of the RTI item bank on risk of bias and precision of observational studies.** *Journal of Clinical Epidemiology* 2012, **65:**163-178.
